# Assessment of radiographic progression in the spines of patients with ankylosing spondylitis treated with adalimumab for up to 2 years

**DOI:** 10.1186/ar2794

**Published:** 2009-08-24

**Authors:** Désirée van der Heijde, David Salonen, Barbara N Weissman, Robert Landewé, Walter P Maksymowych, Hartmut Kupper, Shaila Ballal, Eric Gibson, Robert Wong

**Affiliations:** 1 Department of Rheumatology, C1R, Leiden University Medical Center, PO Box 9600, 2300 RC Leiden, The Netherlands; 2 Department of Medical Imaging, University of Toronto, 600 University Avenue, Toronto, ON M5G 1X5, Canada; 3 Brigham and Women's Hospital, 75 Francis Street, Boston, MA 02115, USA; 4 Department of Internal Medicine/Rheumatology, Maastricht University Medical Center, PO Box 616, 6200 MD Maastricht, The Netherlands; 5 Medicine/Rheumatic Disease Unit, University of Alberta, 562 Heritage Medical Research Building, Edmonton, AB T6G 2S2, Canada; 6 Abbott GmbH & Co. KG, Knollstrasse 50, Ludwigshafen 67061, Germany; 7 Formerly Abbott Laboratories, 300 Interpace Parkway B, Parsippany, NJ 07054, USA

## Abstract

**Introduction:**

Ankylosing spondylitis (AS) is a chronic rheumatic disease associated with spinal inflammation that subsequently leads to progression of structural damage and loss of function. The fully human anti-tumor necrosis factor (anti-TNF) antibody adalimumab reduces the signs and symptoms and improves overall quality of life in patients with active AS; these benefits have been maintained through 2 years of treatment. Our objective was to compare the progression of structural damage in the spine in patients with AS treated with adalimumab for up to 2 years versus patients who had not received TNF antagonist therapy.

**Methods:**

Radiographs from patients with AS who received adalimumab 40 mg every other week subcutaneously were pooled from the Adalimumab Trial Evaluating Long-Term Efficacy and Safety for Ankylosing Spondylitis (ATLAS) study and a Canadian AS study (M03-606). Radiographic progression from baseline to 2 years in the spine of adalimumab-treated patients from these two studies (adalimumab cohort, n = 307) was compared with an historic anti-TNF-naïve cohort (Outcome in AS International Study [OASIS], n = 169) using the modified Stoke AS Spine Score (mSASSS) method.

**Results:**

mSASSS results were not significantly different between the adalimumab cohort and the OASIS cohort, based on baseline and 2-year radiographs. Mean changes in mSASSS from baseline to 2 years were 0.9 for the OASIS cohort and 0.8 for the adalimumab cohort (*P *= 0.771), indicating similar radiographic progression in both groups. When results for patients in the OASIS cohort who met the baseline disease activity criteria for the ATLAS and Canadian studies (OASIS-Eligible cohort) were analyzed, there was no significant difference in mean change in mSASSS from baseline to 2 years between OASIS-Eligible patients and adalimumab-treated patients; the mean changes in mSASSS were 0.9 for the OASIS-Eligible cohort and 0.8 for the adalimumab cohort (*P *= 0.744).

**Conclusions:**

Two years of treatment with adalimumab did not slow radiographic progression in patients with AS, as assessed by the mSASSS scoring system, when compared with radiographic data from patients naïve to TNF antagonist therapy.

**Trial registration:**

Canadian study (M03-606) ClinicalTrials.gov identifier: NCT00195819; ATLAS study (M03-607) ClinicalTrials.gov identifier: NCT00085644.

## Introduction

Ankylosing spondylitis (AS) is a chronic rheumatic inflammatory disease of the axial skeleton, large peripheral joints, and entheses. AS is a member of the spondyloarthritides, a group of disorders that share common clinical, serologic, radiographic, and genetic features, including enthesitis, presence of the human leukocyte antigen-B27 antigen, and radiographic progression that may restrict spinal mobility and potentially evolve into complete spinal ankylosis [1]. Tumor necrosis factor (TNF), a proinflammatory cytokine, is present in biopsies of sacroiliac joints of patients with active disease, suggesting TNF involvement in the inflammatory process of AS [2].

The TNF antagonists etaneracept [3] and infliximab [4] have been shown to reduce the signs and symptoms of active AS and improve disease-related quality of life. In the Adalimumab Trial Evaluating Long-Term Efficacy and Safety for Ankylosing Spondylitis (ATLAS) and Canadian AS (M03-606) studies, adalimumab also demonstrated a reduction in signs and symptoms and improvement in disease-related quality of life in patients with active AS [5,6]; these benefits were maintained over 2 years of treatment [7].

Treatment with adalimumab [8], etanercept [9,10], and infliximab [11] has also been shown to reduce inflammatory activity, inhibiting the progression of radiographic damage in rheumatoid arthritis (RA) and psoriatic arthritis (PsA). *In vitro *and *in vivo *models indicate that the bone destruction is mediated by TNF activation of osteoclasts [12-14].

Although TNF antagonists are effective in treating the signs and symptoms of AS, a clear relationship between TNF and spinal bone formation in patients with AS has not been established. Recently published studies have reported that neither etanercept [3] nor infliximab [4] inhibits structural progression in the spine of patients with AS after 2 years of treatment, suggesting that osteoproliferation in AS is independent of TNF. To further assess the relationship between TNF and spinal bone formation with adalimumab, we compared the radiographic progression in patients with AS treated with adalimumab for 2 years with that of TNF antagonist-naïve patients in a separate historical control group previously treated with conventional nonbiologic therapy.

## Materials and methods

### Patients and study design

#### Adalimumab cohort

Data from the ATLAS and the Canadian AS trials were combined to provide a database of adalimumab-treated patients for the analysis of 2-year radiographic data. ATLAS was a phase III, placebo-controlled, double-blind, randomized, multicenter study conducted in the US and Europe that was designed to demonstrate the safety and efficacy of adalimumab in the treatment of patients with active AS who had an inadequate response or intolerance to one or more nonsteroidal anti-inflammatory drugs (NSAIDs) and who may have additionally failed one or more disease-modifying antirheumatic drugs (DMARDs) [5]. Overall, 315 patients were enrolled in ATLAS. Patients were randomly assigned in a 2:1 ratio to receive either 40-mg adalimumab every other week (eow) subcutaneously (SC) or placebo during a 24-week, placebo-controlled, double-blind period. The 24-week, placebo-controlled period of the study was followed by an open-label extension period during which patients received 40-mg adalimumab eow SC for up to 236 weeks. This study had coprimary endpoints to evaluate the effect of adalimumab on the reduction of signs and symptoms and to assess the inhibition of progression of structural damage in the spine as measured by the mean change in the modified Stoke AS Spine Score (mSASSS) (range 0 to 72) from baseline to 2 years [15].

A smaller AS study conducted in Canada (M03-606) was similar in design and shared the same endpoints as the ATLAS study; a total of 82 patients were enrolled [6]. Data from the 315 patients from ATLAS were pooled with the 82 patients from the Canadian study as a potential source of radiograph data for the primary analysis (n = 397). The ATLAS and Canadian studies were performed with approval from the local ethics committees of the involved centers, and signed informed consent was obtained from all study participants.

#### Historical control cohort

For ethical reasons, a 2-year, placebo-controlled study could not be performed, and therefore radiographic progression in adalimumab-treated patients was compared with radiographic progression in a historical control cohort of TNF antagonist-naïve patients. Established as a prevalence cohort in 1996, the Outcome in AS International Study (OASIS) cohort consists of 217 consecutive Dutch, French, and Belgian patients with AS [16]. The OASIS cohort is representative of patients with AS in rheumatology practice. These patients were treated primarily with NSAIDs, and approximately 10% received treatment with DMARDs. All patients were TNF antagonist-naïve. Because some patients were lost to follow-up, pairs of baseline and 2-year radiographs were available for 186 patients.

#### Primary analysis set

The primary analysis set contained all patients in the OASIS and adalimumab-treated cohorts who had baseline and 2-year radiographs. The primary analysis excluded patients with total spinal ankylosis (TSA), defined as a baseline mSASSS value of 72 (the maximum score). Patients with TSA were excluded from the primary analysis because they could not experience any further radiographic progression. A minimum cutoff of 1.5 years was chosen to maximize the number of adalimumab-treated patients who could be included for evaluation; because the first 24 weeks of both adalimumab studies were randomized and placebo-controlled, patients enrolled for 2 years might have experienced only 18 months of adalimumab exposure. A total of 169 patients from the OASIS study (OASIS cohort) and 307 patients from the adalimumab studies (adalimumab cohort) qualified for the primary analysis set.

#### Secondary (OASIS-Eligible) analysis set

The OASIS-Eligible set included patients in the OASIS cohort who met the eligibility criteria for baseline disease activity as defined in the ATLAS and Canadian studies. A total of 77 patients from the OASIS cohort qualified for the OASIS-Eligible set; this set was compared with the adalimumab cohort in a separate analysis.

### Assessment of radiographic progression

Baseline and 2-year radiographs of the lateral cervical and lumbar spine in patients in the OASIS and the adalimumab cohorts were scored using the mSASSS scoring method [17,18]. Radiographs from the OASIS cohort and the adalimumab cohort were combined, randomized, and read by two independent assessors who were blinded to the origin of cohort, treatment allocation, and sequence. Two readers and one adjudicator were selected based on their experience with musculoskeletal imaging and experience in reading spinal imaging studies of patients with AS. However, none of the assessors was familiar with the OASIS films. The assessors read the radiographs remotely using work stations and a proprietary Computer-Assisted Masked Reading system (CAMR™) (Bio-Imaging Technologies, Inc., now part of BioClinica, Newtown, PA, USA). Radiographic progression was based on the average change in mSASSS of the two assessors over 2 years. If the 2-year mSASSS progression scores of readers 1 and 2 differed by at least 5 mSASSS units for a patient's radiographs, the films were reread by the same readers. The adjudicator evaluated patient radiographs if the discrepancy of at least 5 mSASSS units between readers 1 and 2 persisted following the reread procedure.

### Statistical analysis

The sample size needed was based on the assumption that 80% of patients would have evaluable radiographic x-ray films at year 2. Thus, we expected approximately 150 patients randomly assigned to adalimumab to have been available for radiographic evaluation. In addition, we anticipated having approximately 170 patients from the historical control database. A two-group ranked analysis of variance with a 0.05 type I error was employed with at least 85% power to detect the difference between an adalimumab mean of 0.2 and a historical control mean of 1.2, a difference in means of 1.0, with the assumption of a common standard deviation of 2.8. This calculation took into account possible missing radiographs.

Demographics and baseline characteristics among the randomized treatment groups were summarized and compared. Continuous demographic variables were described by statistical characteristics (for example, number of observations, mean, two-sided 95% confidence intervals, standard deviation, minimum, first quartile, median, third quartile, and maximum) and analyzed using analysis of variance. Discrete demographic variables described by statistical characteristics (for example, frequency tabulations, counts, and percentages) were analyzed using the Fisher exact test.

The primary efficacy analysis compared radiographic progression between the adalimumab cohort and the OASIS cohort using an analysis of covariance model. The primary endpoint, the mean change in mSASSS values from baseline to year 2, was the dependent variable, with cohort as a factor and baseline mSASSS values as a covariate. The primary analysis was performed on the non-TSA patient population. Cumulative probability plots were generated for the change in mSASSS values from baseline to year 2 of adalimumab treatment. The probability of any radiographic progression was modeled as a function of the change from baseline to year 2 in the mSASSS value using an ordinal logistic regression model.

Secondary analyses included comparison of the OASIS-Eligible set with the adalimumab cohort, assessment of correlations between radiographic progression and clinical measures of disease activity, and sensitivity analyses. Several sensitivity analyses were performed to assess the impact of different missing data imputations on the results of the analysis.

Intra- and inter-reader reliability was evaluated using the intraclass correlation coefficient (ICC) for baseline and year-2 radiographs. The ICC is derived from the variance components of the linear model corresponding to the structure of the repeated scoring of the radiographs. Decreased variability is indicated by greater ICC values (range 0 to 1).

## Results

### Baseline demographic and disease characteristics

Significantly different baseline demographic and disease characteristics were observed between the OASIS and adalimumab cohorts (Table 1). Baseline disease activity was significantly lower in the OASIS cohort compared with the adalimumab cohort, as assessed by the Bath AS Disease Activity Index (BASDAI), Bath AS Functional Index (BASFI), total back pain, inflammation, C-reactive protein, and the Patient's Global Assessment of disease activity. Adalimumab-treated patients also had significantly greater mSASSS values at baseline compared with OASIS patients. However, baseline clinical characteristics in the OASIS cohort have been shown not to be predictive of radiographic progression [19]. When used for covariate adjustment in statistical models, baseline variables had no effect on the end result of radiographic progression (data not shown).

**Table 1:** Baseline demographic and disease characteristics

**Demographic characteristic**	**OASIS**		**Adalimumab**		***P *value^a^**
			
	**Number**	**Baseline assessment**	**Number**	**Baseline assessment**	
Age, years	168	43.6 ± 12.7	307	41.8 ± 11.5	0.101
Male, percentage	169	69.2	307	76.5	0.102
Weight, kg	157	72.7 ± 12.8	307	80.0 ± 16.3	< 0.001
Height, cm	161	171.0 ± 9.3	306	172.9 ± 9.5	0.037
Concomitant medications, percentage					
NSAIDs	169	77.5	307	88.3	
DMARDs	169	9.5	307	21.8	
Systemic glucocorticoids	169	1.8	307	9.8	
Disease characteristic					
Disease duration, years	163	11.3 ± 8.7	307	11.2 ± 9.3	0.946
BASDAI, 0-10	166	3.4 ± 2.1	307	6.2 ± 1.7	< 0.001
BASFI, 0-10	158	3.1 ± 2.4	307	5.3 ± 2.1	< 0.001
Total back pain, 0-10	166	3.5 ± 2.4	307	6.7 ± 1.9	< 0.001
Inflammation, 0-10^b^	167	3.4 ± 2.6	307	6.7 ± 2.0	< 0.001
Patient's Global Assessment of disease activity, 0-10	165	3.7 ± 2.7	306	6.4 ± 2.0	< 0.001
C-reactive protein, mg/dL^c^	160	1.5 ± 1.9	302	1.9 ± 2.5	0.036
mSASSS, 0-72	169	15.8 ± 17.6	307	19.8 ± 19.3	0.028

Values are mean ± standard deviation unless otherwise noted. ^a^*P *values calculated using one-way analysis of variance or the Fisher exact test. No statistical comparison of concomitant medications was completed. ^b^Mean of questions 5 and 6 of the Bath Ankylosing Spondylitis Disease Activity Index (BASDAI). ^c^Using the ultrasensitive assay (normal range, 0.007 to 0.494 mg/dL). BASFI, Bath Ankylosing Spondylitis Functional Index; DMARD, disease-modifying antirheumatic drug; mSASSS, modified Stoke Ankylosing Spondylitis Spinal Score; NSAID, nonsteroidal anti-inflammatory drug; OASIS, Outcome in Ankylosing Spondylitis International Study.

A total of 169 patients from the OASIS study (OASIS cohort) and 307 patients from the adalimumab studies (adalimumab cohort) qualified for the primary analysis set. At the time of this analysis, the 307 adalimumab-treated patients had been treated for at least 78 weeks (approximately 1.5 years). Their mean adalimumab dosage was 45.6 mg eow. Ninety patients of the original 397 in the adalimumab cohort were excluded from the analysis because they had TSA or fewer than 1.5 years of total exposure to adalimumab.

### Primary mSASSS analysis

**Figure 1:**
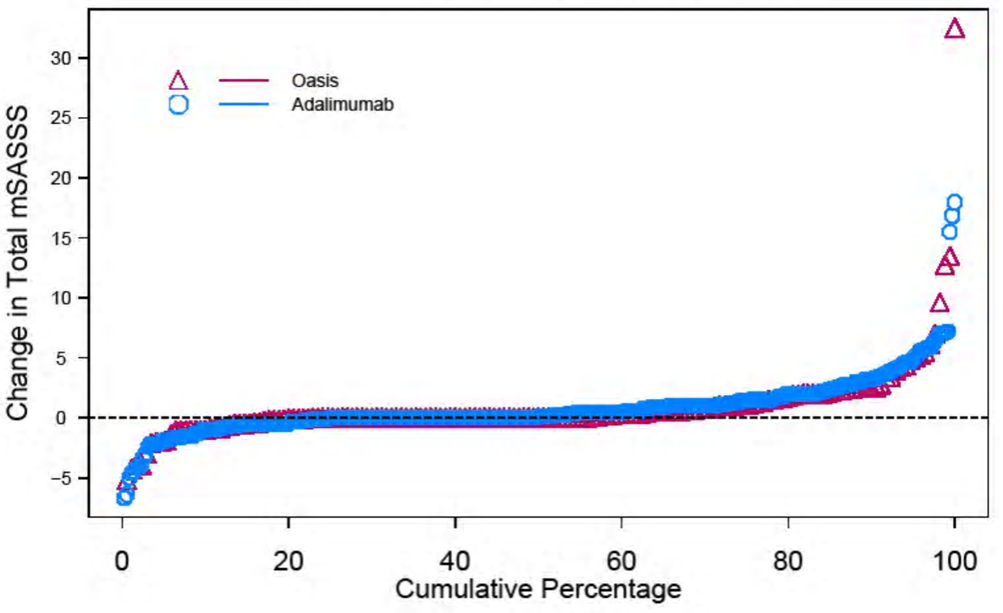
Probability plot of 2-year progression in the modified Stoke Ankylosing Spondylitis Spine Score (mSASSS). The cumulative probability plot illustrates the change in mSASSS values from baseline to 2 years in the adalimumab cohort (n = 307) and OASIS (n = 169) cohort (patients without total spinal ankylosis). In both cohorts, over 40% of the patients showed some change and about 10% of the patients showed a change of at least 5 in mSASSS from baseline to year 2. No significant differences between the adalimumab and OASIS cohorts were observed. OASIS, Outcome in Ankylosing Spondylitis International Study.

No significant difference in radiographic progression, as assessed by the mean change in mSASSS from baseline to year 2, was observed between the OASIS cohort and the adalimumab cohort (Table 2 and Figure 1). More than 40% of the patients in both cohorts experienced a change in mSASSS from baseline to year 2 (Figure 1).

**Table 2:** mSASSS results for primary analysis set and for OASIS-Eligible patients

**Cohort**	**Number**	**Mean change ± SD from baseline to year 2**	***P *value^a^**
Primary mSASSS analysis set			
OASIS	169	0.9 ± 3.3	0.771
Adalimumab	307	0.8 ± 2.6	
OASIS-Eligible patients			
OASIS	77	0.9 ± 4.1	0.744
Adalimumab	307	0.8 ± 2.6	

^a^*P *value calculated using analysis of covariance model with therapy as a factor and baseline modified Stoke Ankylosing Spondylitis Spinal Score (mSASSS) as a covariate. OASIS, Outcome in Ankylosing Spondylitis International Study; SD, standard deviation.

### OASIS-Eligible set

There was no significant difference in the mean change in mSASSS from baseline to year 2 between the adalimumab cohort and OASIS-Eligible patients (that is, patients in the OASIS cohort who met all baseline disease activity criteria for the ATLAS and Canadian studies) (Table 2). The mean change in mSASSS for the OASIS-Eligible cohort did not differ from the mean change in mSASSS for the full OASIS cohort (0.9 ± 3.3 versus 0.9 ± 4.1, respectively).

### Sensitivity analyses

A number of sensitivity analyses were conducted to assess factors that could potentially affect the results of the primary analysis. None of the sensitivity analyses revealed a significant difference in radiographic progression between the adalimumab cohort and the OASIS cohort (data not shown). For example, a sensitivity analysis excluding the bottom C7 and T1 top vertebral sites (which are often difficult to read owing to obscuring of the C7 and T1 views by the shoulders on lateral cervical films) did not change the results of the primary analysis, nor did sensitivity analyses exploring alternate imputations for missing vertebral sites.

### Reader reliability

Intra- and inter-reader reliability was evaluated using the ICC. Intrareader reliability testing was based on 56 patients (approximately 10%) from OASIS, ATLAS, and the Canadian AS study combined. The ICC values for reader 1 were 0.982 for baseline radiographs and 0.987 for year-2 radiographs. For reader 2, the ICC values were 0.913 for baseline radiographs and 0.931 for year-2 radiographs. Intrareader reliability for the change in mSASSS from baseline to year 2 was lower for reader 1 (ICC = 0.319) than for reader 2 (ICC = 0.810) because the intrareader analysis was conducted using only 10% of the radiographs and this figure was heavily influenced by one outlier for reader 1. The ICC for inter-reader variability for the change in mSASSS was 0.673. In total, of the 550 radiograph cases read, 19 (3%) were adjudicated.

### Radiographic progression and clinical measures of disease activity

Another assessment evaluated whether changes in mSASSS values from baseline to year 2 in the adalimumab cohort were correlated with clinical measures of disease activity at baseline or changes from baseline in clinical measures of disease activity. Changes in mSASSS were correlated with baseline scores on several clinical outcome measures, including the Bath AS Metrology Index (BASMI), BASFI, and short-form 36 health survey (SF-36) physical component summary (PCS). However, there was no significant correlation between change in radiographic progression and change from baseline for any of the following clinical measures: BASDAI, BASMI, BASFI, C-reactive protein, and SF-36 PCS for all patients and matrix metalloproteinease-3 (MMP-3) (n = 37) and urinary type II collagen C-telopeptide (n = 38) for patients in the Canadian study (data not shown).

## Discussion

In the present study, radiographic progression in patients with AS treated with adalimumab 40 mg eow was compared with radiographic progression in patients in the OASIS historical control group. There was no difference between the adalimumab and OASIS cohorts in the mean change in mSASSS from baseline to year 2 based on the primary efficacy analysis of patients' radiographs. Similarly, no difference between the adalimumab cohort and the OASIS cohort was observed when the analysis included only the subset of OASIS patients (OASIS-Eligible set) who satisfied the minimum baseline disease activity requirements of the adalimumab studies. Additional sensitivity analyses were performed to investigate other factors that could have potentially affected the results (for example, vertebral imputation), but these analyses did not reveal significant differences in radiographic progression between adalimumab-treated patients and the control cohort.

Intra- and inter-reader reliability was evaluated using ICC values. In AS studies, ICC values generally range from 0.6 to 0.7 [20]; ICC values in the present study were within expected ranges and did not contribute to the negative results.

The OASIS and adalimumab cohorts were heterogeneous with respect to baseline demographic and disease characteristics. Adalimumab-treated patients had significantly greater disease activity and mSASSS values compared with the OASIS cohort. In addition, a greater percentage of adalimumab-treated patients were taking NSAIDs at baseline. NSAID therapy has been reported to inhibit syndesmophyte formation and structural progression of AS; however, this finding needs to be confirmed [21]. Differences in baseline characteristics between the adalimumab and control cohorts had no apparent effect on radiographic progression. A more direct and stringent comparison between adalimumab and control cohorts would ideally be performed in a randomized, double-blind, placebo-controlled trial. However, owing to the rapid effectiveness of TNF antagonists in the treatment of AS, it would be unethical to conduct a 2-year, placebo-controlled trial to assess radiographic progression. Thus, the historical control OASIS cohort is the best available comparator for adalimumab-treated patients.

Radiographic progression in patients with AS has been reported with TNF antagonists etanercept and infliximab [3,4]. As in the present study of adalimumab, these studies evaluated changes in mSASSS from baseline to year 2 of treatment and used the OASIS historical control group for comparison. Baseline characteristics and radiographic progression results in these studies and those of the present study of adalimumab were similar (Table 3). The similar results of the three independent cohorts of patients treated with TNF antagonists, as well as the similar results of the OASIS cohort scored three times independently, are striking, especially if one takes into account the fact that each study employed a different pair of readers. Thus, the obtained results are based on scores of six different readers.

**Table 3:** Comparison of 2-year radiographic progression among tumor necrosis factor antagonists

		**Baseline characteristics**		**Radiographic progression results**	
			
**Cohort**	**Number^a^**	**Disease duration, years^b^**	**mSASSS value^b^**	**Mean mSASSS change from baseline to year 2^b^**	**Between-cohort *P *value**
Etanercept [2]	257	10 ± 8.5	16 ± 18.3	0.9 ± 2.5	0.996
OASIS	175	11 ± 8.5	14 ± 17.6	1.0 ± 3.2	
Infliximab [3]	156	10.2 ± 8.7	17.7 ± 17.9	0.9 ± 2.6	0.541
OASIS	165	11.3 ± 8.6	15.8 ± 18.1	1.0 ± 3.2	
Adalimumab	307	11.2 ± 9.3	19.8 ± 19.3	0.8 ± 2.6	0.771
OASIS	169	11.3 ± 8.6	15.8 ± 17.6	0.9 ± 3.3	

^a^Number of patients with a baseline and a year-2 radiograph. ^b^Values are mean ± standard deviation. mSASSS, modified Stoke Ankylosing Spondylitis Spinal Score; OASIS, Outcome in Ankylosing Spondylitis International Study.

It is unclear why TNF antagonist therapy does not appear to inhibit radiographic progression in patients with AS. Given the insidious nature of spinal ankylosis, the 2-year timeframe of the studies may have been insufficient to fully assess radiographic damage and progression. There is one small study suggesting that infliximab slowed the progression of structural damage from 2 to 4 years of therapy [22]. However, that study had notable limitations, including differences in baseline disease activity (that is, BASDAI scores) between patients receiving infliximab versus traditional therapies (OASIS), and differences in scoring methods [22]. Therefore, this effect requires further investigation. It is possible that inhibition of radiographic progression may take even longer periods of continuous TNF antagonist therapy [23].

Initiation of anti-TNF therapy in patients with very early AS or preradiographic spondyloarthritis may prevent radiographic progression in the spine, but there are no data as of yet to confirm this hypothesis. The studies of radiographic progression in patients treated with etanercept, infliximab, or adalimumab included patients with long-standing AS and evidence of at least grade 2 sacroiliitis (satisfying the modified New York criteria [24]). Therefore, studies in patients with spondyloarthritis with preradiographic sacroiliitis or early evidence of sacroiliitis (and who do not yet satisfy the modified New York criteria) may be more likely to demonstrate inhibition of structural damage following TNF antagonist therapy. Adalimumab has been shown to significantly suppress serum concentrations of MMP-3, a biomarker that is a significant independent predictor of structural damage progression in AS [25,26]. However, we found no significant correlation between change in radiographic progression and change from baseline in concentrations of MMP-3 for adalimumab patients in the Canadian study, which may reflect the small sample size. Longer-term studies will be needed to further assess the full impact of TNF antagonist therapy on radiographic progression.

One possible limitation of this study may be the use of the mSASSS scoring system for quantification of disease progression. This system is limited in that it takes into account the structural changes in the vertebral bodies and related soft tissues of the cervical and lumbar spine without evaluating possible further damage at the posterior elements of the cervical and lumbar spine, the thoracic spine, or the facet joints [20]. However, the mSASSS has been validated for AS and is currently the standard method for scoring radiographic progression [15,20]. Moreover, because this method was used in the analysis of both the adalimumab and the OASIS cohorts, it is doubtful that the results of this study were influenced by the mSASSS scoring method. Moreover, the method was able to detect changes for more than 40% of the patients.

TNF is associated with inflammation and bone destruction in RA and PsA. TNF antagonists have been shown to reduce disease activity and inhibit the degenerative bone processes in RA and PsA [8-11]. In contrast, TNF antagonists have not been shown to inhibit the bone formation associated with AS despite amelioration of the signs and symptoms of the disease. Consistent with this observation, uncoupling of inflammation and bone formation has been reported in animal models of spondyloarthritis [27-29]. Recent evidence suggests that new bone formation may be more likely to occur at the sites of spinal inflammation in patients with AS; two studies reported that more syndesmophytes developed at inflamed vertebral edges than at noninflamed vertebral edges, although the majority of syndesmophytes developed at vertebral edges without inflammation at baseline [30,31]. However, one study demonstrated the development of new syndesmophytes even when inflammation had resolved after anti-TNF therapy [31]. It has been proposed that each AS patient is likely to have several spinal lesions at different stages of evolution. In addition, it may be possible that very early lesions resolve with anti-TNF therapy prior to the induction of reparative changes, whereas for more mature inflammatory lesions, reparation is allowed to proceed following resolution of inflammation with anti-TNF therapy. The overall outcome for the individual patient is then little change at the level of the entire spine [32]. The observation that new syndesmophytes also develop where there appeared to have been no prior inflammation at vertebral corners also points to the possibility of non-inflammation-driven pathways of reparation [33,34]. Further research is needed in this area.

## Conclusions

In patients with long-standing AS, 2 years of treatment with adalimumab was effective in improving axial symptoms and reducing spinal inflammation but did not significantly inhibit radiographic progression. These findings are consistent with those reported with etanercept and infliximab. Additional studies that examine longer-term data with TNF antagonists and earlier use of TNF antagonists to inhibit inflammation and syndesmophyte formation are needed to better understand the relationship between chronic inflammation and spinal ankylosis in AS.

## Abbreviations

AS: ankylosing spondylitis; ATLAS: Adalimumab Trial Evaluating Long-Term Efficacy and Safety for Ankylosing Spondylitis; BASDAI: Bath Ankylosing Spondylitis Disease Activity Index; BASFI: Bath Ankylosing Spondylitis Functional Index; BASMI: Bath Ankylosing Spondylitis Metrology Index; DMARD: disease-modifying antirheumatic drug; eow: every other week; ICC: intraclass correlation coefficient; MMP-3: matrix metalloproteinease-3; mSASSS: modified Stoke Ankylosing Spondylitis Spine Score; NSAID: nonsteroidal anti-inflammatory drug; OASIS: Outcome in Ankylosing Spondylitis International Study; PCS: physical component summary; PsA: psoriatic arthritis; RA: rheumatoid arthritis; SC: subcutaneously; SF-36: short-form 36 health survey; TNF: tumor necrosis factor; TSA: total spinal ankylosis.

## Competing interests

HK is an employee of an affiliate of Abbott Laboratories (Abbott Park, IL, USA) and own shares of Abbott stock. SB, EG, and RW were employees of Abbott Laboratories at the time the analyses were completed and own shares of Abbott stock. The Maastricht University Medical Center was financially supported for use of the OASIS database and the readers for reading of blinded radiographs by Abbott Laboratories. DvdH has received consulting fees, research grants, and/or speaking fees from Abbott Laboratories, Amgen (Thousand Oaks, CA, USA), sanofi-aventis (Paris, France), Bristol-Myers Squibb Company (Princeton, NJ, USA), Centocor, Inc. (Horsham, PA, USA), Pfizer Inc (New York, NY, USA), Roche (Basel, Switzerland), Schering-Plough Corporation (Kenilworth, NJ, USA), UCB (Brussels, Belgium), and Wyeth (Madison, NJ, USA). DS and WPM have received consulting fees, speaking fees, and/or research grants from Abbott Laboratories, Amgen, sanofi-aventis, Pfizer Inc, Schering-Plough Corporation, and Wyeth. RL has received consulting fees, research grants, and/or speaking fees from Abbott Laboratories, Amgen, Bristol-Meyers Squibb Company, Centocor, Inc., Pfizer Inc, Schering-Plough Corporation, UCB, and Wyeth. BNW declares that she has no competing interests.

## Authors' contributions

SB and EG designed and performed the statistical analyses. DS and BNW performed the blinded reading of the radiographs. RL was an investigator for the OASIS study and was the adjudicator for the radiographic reads. DvdH was the principal investigator of the OASIS study and is the principal investigator who assisted in designing the ATLAS study. WPM is the principal investigator and assisted in designing the Canadian M03-606 study. RW and HK assisted in designing the ATLAS and Canadian studies and coordinated the radiographic reads with both Bio-Imaging Technology, Inc. (now part of BioClinica, Newtown, PA, USA) and independent readers. All authors read and approved the final manuscript.
